# Does the Registered Dentists’ Program Alleviate the Socioeconomic Gap in the Use of Dental Sealants?

**DOI:** 10.3390/ijerph17217828

**Published:** 2020-10-26

**Authors:** Ji-Eun Jeon, A-Rang Lim, Hyang-Ah Park, Jae-In Ryu

**Affiliations:** 1 Department of Preventive and Social Dentistry, Graduate School, Kyung Hee University, Seoul 02447, Korea; jjelove11@gmail.com (J.-E.J.); arang0720@gmail.com (A.-R.L.); qkrgiddk12@gmail.com (H.-A.P.); 2 Department of Preventive and Social Dentistry, College of Dentistry, Kyung Hee University, Seoul 02447, Korea

**Keywords:** healthcare disparities, pit and fissure sealants, socioeconomic factors, child, oral health

## Abstract

Many countries provide preventive dental care for children to reduce inequalities. In Korea, the registered dentists’ program was implemented to promote oral health and prevent oral diseases in primary school students. This study aimed to evaluate the registered dentist program through the sealant utilization rate using national cohort data and to compare the socioeconomic gap of the cohorts by participation. The sample cohort data were obtained from the National Health Insurance Sharing Service (NHISS) in South Korea. The utilization of dental sealants was analyzed using the chi-square test according to the independent variables of each year. To identify the independent effects of participation in the registered dentists’ program, a panel logistic regression analysis of the utilization of dental sealants was performed. The participants were 1.35 times more likely to have dental sealants than non-participants. The significance of income quintiles disappeared in the case groups. The gap became more obvious in the employees of the control group even after adjusting for all variables. Implementing oral health programs can alleviate inequality with a relative increase in utilization in vulnerable populations.

## 1. Introduction

During the childhood period, oral health and related behaviors are crucial because they affect growth and development until adulthood [1]. Dental caries is the most common chronic disease in children and it is a global burden of disease and a public health challenge [2,3]. There are various risk factors, for example, oral health behaviors including tooth brushing to remove dental plaque and to improve the dental hygienic environment [4,5,6]. Oral health management reduces dental costs in the long term [7] and proper care for dental caries increases the growth rate and improves the quality of life [8]. However, socioeconomic position (SEP) is an important factor in determining oral health status [2,9,10,11] and dental treatment in children [12]. Oral disease is described as “a sensitive clinical marker of disadvantage, being an early indicator of population ill health” [2]. Thus, it is necessary to concentrate on promoting oral health in an upstream approach for oral health equity [13]. There was evidence that the provision of preventive services for oral health, for example, dental sealants [14,15,16], fluoride application [17,18], and education [19,20], relieved the socioeconomic disparity. The effectiveness of dental sealants has been proven by several systematic reviews over several years [21,22,23,24]. They concluded that resin-based sealants reduced caries by between 11% and 51% compared to no sealant. The long-term effect of intensive prevention on dental health in primary school children showed that there were no clear SEP differences in the intervention group and dental sealants were the most important factor for caries prevention [25]. In the U.S., the service use of sealant increased faster in poor children than in those who were not due to public dental sealant programs and community health centers [26,27].

Many countries have tried to provide preventive dental care for children to reduce such inequalities. This is “knowledge translation”, which is defined as “the synthesis, exchange, and application of knowledge by relevant stakeholders to accelerate the benefits of global and local innovation in strengthening health systems and improving people’s health” [28,29]. In the United States, dental screening, prevention, and treatment services are provided in the form of the Early and Periodic Screening, Diagnosis, and Treatment (EPSDT) program by Medicaid and the Children’s Health Insurance Program (CHIP) [30]. Canada has a Healthy Smiles program that provides dental care cards to low-income children and adolescents for oral healthcare services [31]. France has a national oral health prevention program called “M’T Dents” for children and adolescents aged 6, 9, 12, 15, or 18 years old [32]. The program provides an oral examination, oral hygiene advice for children and their parents, and fillings and preventive care if necessary. In Scotland, “Childsmile” operates from birth to nursery or school to improve oral health from infancy and reduce inequalities in access to dental care [33]. The poorest schools within an area receive fluoride varnish twice a year. Dental sealants are also provided through family dentist registration.

The oral health status of children and adolescents is gradually improving with the daily use of fluoride toothpaste, professional fluoride application, and dental sealants in Korea [34]. However, dental caries is still the most frequent disease in children and there are inequalities in oral health due to socioeconomic status [35,36,37]. In 2012, the city of Seoul introduced a registered dentist program in coordination with compulsory annual oral examinations in schools. A pilot project for 4th grade students in primary school was initiated. Six districts were selected among twenty-five in the city and the project was conducted for 3 years, starting in 2012. The program encompassed dental examinations, education, and preventive services. The package was provided in a local clinic under collaboration. The dentists received forty dollars per person by the city government and the children paid no charge at the point of use. Dental examination included screening, Patient Hygiene Performance (PHP) index, and panoramic radiography. Oral health education consisted of ways to manage oral hygiene, eating habits, tooth brushing, and flossing. Preventive treatment covered oral prophylaxis, professional fluoride application, and dental sealant [38]. Some studies evaluated the registered dentists’ program with extended coverage of treatment for low-income children [39,40,41] but they were limited in the study population and had a lack of representative samples.

This study aimed to evaluate the registered dentist program through the sealant utilization rate using national cohort data and to compare the socioeconomic gap of the cohorts by participation.

## 2. Materials and Methods

### 2.1. Study Subjects

This study used sample cohort data from 2002 to 2015 provided by the National Health Insurance Sharing Service (NHISS) in South Korea. The service provided the sample research database (DB), which is composed of a sample cohort DB, medical checkup DB, elderly cohort DB, working women cohort DB, and infant medical checkup DB [42]. The sample research DB refers to the data standardized in a shareable form by extracting the sample to reduce the limited access due to large size and personally identifiable information issues. The DB allows long-term observation of the same individuals as a cohort and connects qualification data, including socioeconomic variables (location of residence, month and date of death, cause of death, income rank, etc.) to treatment details, medical checkup data, and the clinic to enable the analysis of a causal or temporal relationship. This sample cohort included approximately one million qualified individuals extracted from Medicare recipients and National Health Insurance (NHI) subscribers in 2006, which was 2% of the total population.

The study population included children who lived in Seoul and were born in 2002 and primary school fourth graders in 2012. From this population, students who lived in six districts and participated in the registered dentist program comprised the study cohort and the control was those who did not participate. The study period considered both the extended coverage and implementation of the projects. The duration of the study analysis was set from 2011 to 2015, including a year before and after the pilot projects to check the yearly change in utilization in the study cohort. Finally, the total number of study subjects was 1785 in 2011. This study was granted an exemption from the institutional review board designated by the Ministry of Health and Welfare in South Korea (P01−201907−21−033).

### 2.2. Study Variable

Dental sealant was selected as the dependent variable for this study. Among the services provided through the registered dentists’ program, sealant is one of the most effective preventive measures for dental caries, especially in children and adolescents [43]. In addition, sealant was the only service to be covered and claimed by the National Health Insurance Service (NHIS) in Korea. The coverage of sealant began in December 2009, limited to the first molar for children aged 6−14 years. In 2012, coverage was expanded to the second molars and even to children under age 6. One year later, it was expanded further, to children up to 18 years old. In 2017, the rate of out-of-pocket payments decreased from 30% to 10% [44].

Independent variables were gender, type of subscription, income quintile, and participation. The type of subscription was classified as Medicare and health insurance. The children were usually dependent on householders and the qualification was assigned by the householder’s position, whether they were self-employed or employees. The income quantile was analyzed separately by the type of subscription because it was based on a different contribution system. The contribution of a self-employed insurer is based on the household’s wealth, such as income, property, and car ownership. In the case of an employee, the contribution is set by the employee’s income, including wage. The registered dentists’ project participants were the study cohort who resided in six selected districts and the remainder, from 19 districts, were the control group who did not participate in the project.

### 2.3. Statistical Analysis

The cohort data consisted of four types of DBs, qualification, treatment, medical checkup, and clinic. It contained socioeconomic variables, the status of medical resource utilization, and the clinic. The final data set for this study was created by merging the DBs with the unidentifiable category of individuals. The utilization of dental sealants was examined using a chi-square test, according to the independent variables of each year. To identify the independent effects of participation in the registered dentists’ program, a panel logistic regression analysis of the utilization of dental sealants was performed. The results were presented after separation by study group and control. The variables were included in the analysis step-by-step to evaluate the effect on the dependent variable. All study samples were analyzed with an unadjusted model: Model 1, adjusted for gender and insurance type or income quintile; and Model 2, adjusted for gender, insurance type or income quintile, and participation. The variables that were significant at a *p*-value of less than 0.05 were determined to have significant effects on the dependent variable of dental sealant utilization. Data analysis was carried out using the STATA version 15.1 statistical software package (StataCorp, College Station, TX, USA).

## 3. Results

There were 1785 students in this study sample cohort (Table 1). There were always more boys than girls during the study period. Medicaid beneficiaries were very few, no more than two percent. Less than three-quarters of the students were dependent on employees’ health insurance, and half of them were from the highest income. The number of students who were pilot program participants was 411 in 2011, a quarter of the total. The utilization rate of dental sealants in the study population ranged from 9.4% to 17.4%. The rate reached its highest level at 17.4% in the last year of the program. There was a statistically significant difference in income only within the self-employed in 2012. The utilization rate of dental sealants was almost double in the cohort participants compared to the control group, especially during the pilot period (*p* ≤ 0.01). The results are shown in Table 2, which separates the sample into the cohort and the control groups by participation in the registered dentists’ program. In cohort students, there were no significant differences by gender, insurance type, or income for the utilization rate of dental sealants (all *p* > 0.05). Participants visited the dental clinic and had dental sealants more frequently during the pilot program period, particularly in the lower income group. The utilization rate increased even in the control group, though not as much as that of the cohort participants.

A significant difference by participation was shown in Table 3, even after adjusting all the independent variables in the panel logistic regression model (*p* < 0.001). Participants were 1.35 times more likely to have dental sealants than non-participants. The dependents of the self-employed had a higher gap by participation than the employees, 1.63 vs. 1.26 times, even though both of them showed significant differences. There was also significant differentiation by income quintile. The scale of the gap was larger in the self-employed groups, whereas the range was wider in the employee group. After separating the students by participation in these models, all the significance by variables disappeared in the cohort groups (*p* > 0.05). Meanwhile, the gap by income became more obvious in the employee group of the control even after adjusting for all the variables in Model 1 (Table 4).

## 4. Discussion

The likelihood of using dental sealant was 1.35 times higher for registered dentist program participants. During the pilot period, inequality in utilization of preventive services was not found by income in this group.

The utilization rate of dental sealant significantly increased in cases where the registered dentists’ program was implemented. When a school-based sealant provision project was implemented in the United States, it proved the cost-effectiveness of the service by preventing 485 fillings and the loss of 1.59 disability-adjusted life years (DALYs). The benefits exceeded the costs of targeting high-risk children [26,45,46,47,48,49,50,51]. In a Chinese cohort, the risk of dental caries in children with sealants was 37% lower than that of children without them. After 3 years, the risk decreased by up to 44%, especially in rural areas [52]. A preventive national dental program for children with dental health examination (DHE) improved access and reduced the cost of the National Health Insurance Fund (NHIF) in France (known as M’T Dent) [53]. During the Childsmile program in Scotland, 14,000 elementary students were enrolled in 142 practices or clinics and received 28,000 fluoride varnishes [54,55,56,57,58,59]. Therefore, it was found that a program providing preventive oral health services could improve children’s access to dental services.

There was no significant disparity in the sealant utilization rate by income in the region where the registered dentist program was implemented in Korea. In the registered dentists’ program, the services were provided as compulsory by cooperating with the regional education office. Accessibility to preventive services in the low-income group increased and the inequality might have disappeared as a result. Some researchers have suggested the reason for this alleviation was due to the wider point of contact for service use [26,27]. There was also a suggestion that school-based prevention programs could increase sealant retention for high-risk students [60]. A subsidy for the fee to reduce financial barriers can be another powerful tool to decrease inequalities in children and adolescents [16]. In the British study, the provision of preventive dental care treatment, such as dental sealants or fluoride, eliminated the inequality of dental care accessibility [61]. There was a report that disparities in the treatment of dental caries by household income existed [12]. When the financial barriers to dental service were high, children from the lower-income group had less opportunity to receive treatment than those in the higher-income groups. In this respect, the registered dentists’ program seemed to alleviate income inequality by eliminating financial barriers with no charge and increasing dental access universally even for economically disadvantaged groups.

In the control group where the program was not implemented, there was a significant difference in the sealant utilization rate by income even after all variables were adjusted. In Korea, dental sealants for children and adolescents have been covered by health insurance since 2009, but out-of-pocket payment was 30%, which was almost USD 10 to 20 per tooth during the pilot period. This can be a barrier to dental access for vulnerable populations who seldom use preventive services [62]. There is a need for intervention for children and adolescents who have low access to dental care due to regional inequality in the use of sealants even after insurance coverage [63]. However, even in countries where all dental care for children under the age of 18 is covered by mandatory health insurance free of charge, inequality in oral health status still exists [64]. Some studies explain that vulnerable groups have a higher rate of oral diseases because they purchase and consume cheap processed foods [65], have limited oral health literacy, and continue to have unhealthy behaviors [66]. This results in a worsening of oral health conditions. If this vicious cycle continues, unmet dental care needs increase as well [67]. Inequalities in dental care and oral health affect each other and still exist in many countries. Programs should be conducted to improve accessibility of dental services in the short term, to improve oral health in the long term, and to reduce inequality overall. In the next study, an oral health status survey should be conducted for the regions that participated in the registered dentists’ program and the effectiveness of the program should be evaluated with more diverse details.

This study has several limitations. First, the utilization rate of dental sealants in the first year in the participating group was relatively lower than in other years. Participation was poor at the beginning of the program, as expected. There is one more possibility that emerged from the claimant data. This study used the data claimed through NHIS, which are strong as representative data but they were was not directly acquired from the pilot project. The Metropolitan Government of Seoul did not recommend claiming dental sealants in the first year because this project was financially supported by the city and it can be double charged at the point of service provision. Therefore, there could be a possibility that claims in the first year might be omitted to a degree. This limitation will be solved when a national project is implemented linking with the NHIS claim process. Second, it was difficult to secure a sufficient number of samples because only six districts were selected to implement the registered dentists’ program and the number of children who participated in the project was relatively small because it was a sample cohort, 2% of the total population. When they were separated into the study cohort and the control, the number of children who used dental sealants was not large enough as well. This study used panel analysis to overcome this problem, including the period before and after the pilot project. It is a powerful data set of nationally representative data with insurance claims for the entire population of Korea. It is necessary to conduct a study using more samples and with a longer duration or with the whole population participating in the registered dentists’ program. Lastly, this study analyzed the differences in use of preventive dental services, such as dental sealants. However, it is not guaranteed whether it will extend to equality in oral health status. Social gradients in caries were shown in all components of dental caries experiences as decayed, missing, or filled tooth (DMFT), though the level of untreated caries was higher than others [68]. The Oral Health Promotion (OHP) program increased the effectiveness of prevention but it was not equally influential everywhere, especially in socially deprived areas with dental needs [69]. A social gradient would show a different pattern in the utilization of dental care services, including preventive and conservative treatment, and dental disease status [61]. The long-term effect of preventive service provision needs to be analyzed using the oral health survey within the study cohort in the next study.

## 5. Conclusions

In conclusion, the utilization rate of dental sealants increased as the number of students registered in the dentists’ program increased. The differences in using the service by socioeconomic position were not significant in the participating group. The nonparticipating group showed inequality in preventive services even though there was coverage for the service. The accessibility for children and adolescents of preventive services can be improved through the provision of universal dental services. Implementing oral health programs can alleviate inequality with a relative increase in utilization among vulnerable populations.

## Figures and Tables

**Table 1:** The distribution of study sample cohort with dental sealant in the National Health Insurance Sharing Service (NHISS).

Variables	3rd Grade (2011)				4th Grade (2012)				5th Grade (2013)				6th Grade (2014)				7th Grade (2015)			
	Subject		with Sealant		Subject		with Sealant		Subject		with Sealant		Subject		with Sealant		Subject		with Sealant	
	N	(%)	N	(%)	N	(%)	N	(%)	N	(%)	N	(%)	N	(%)	N	(%)	N	(%)	N	(%)
Total	1785	(100.0)	268	(15.0)	1735	(100.0)	180	(10.4)	1724	(100.0)	243	(14.1)	1710	(100.0)	297	(17.4)	1707	(100.0)	161	(9.4)
Gender																				
Boys	932	(52.2)	144	(15.5)	910	(52.4)	105	(11.5)	897	(52.0)	126	(14.0)	893	(52.2)	134	(15.0) *	895	(52.4)	92	(10.3)
Girls	853	(47.8)	124	(14.5)	825	(47.6)	75	(9.1)	827	(48.0)	117	(14.1)	813	(47.8)	163	(20.0)	812	(47.6)	69	(8.5)
Insurance type																				
Medicaid	26	(1.5)	1	(3.8)	26	(1.5)	4	(15.4)	28	(1.6)	3	(10.7)	31	(1.8)	5	(16.1)	31	(1.8)	3	(9.7)
Self-employed	568	(31.8)	80	(14.1)	522	(30.1)	42	(8.0)	500	(29.0)	68	(13.6)	477	(27.9)	71	(14.9)	463	(27.1)	32	(6.9)
Employee	1191	(66.7)	187	(15.7)	1187	(68.4)	134	(11.3)	1196	(69.4)	172	(14.4)	1202	(70.3)	221	(18.4)	1213	(71.1)	126	(10.4)
Income quintile																				
Self-employed																				
1st	42	(7.4)	8	(19.0)	44	(8.4)	-	(0.0) *	43	(8.6)	4	(9.3)	48	(10.1)	2	(4.2)	54	(11.7)	4	(7.4)
2nd	84	(14.8)	8	(9.5)	80	(15.3)	6	(7.5)	62	(12.4)	6	(9.7)	56	(11.7)	5	(8.9)	50	(10.8)	3	(6.0)
3rd	125	(22.0)	14	(11.2)	118	(22.6)	7	(5.9)	129	(25.8)	18	(14.0)	107	(22.4)	17	(15.9)	105	(22.7)	6	(5.7)
4th	152	(26.8)	26	(17.1)	128	(24.5)	19	(14.8)	123	(24.6)	19	(15.4)	120	(25.2)	20	(16.7)	119	(25.7)	6	(5.0)
5th	165	(29.0)	24	(14.5)	152	(29.1)	10	(6.6)	143	(28.6)	21	(14.7)	146	(30.6)	27	(18.5)	135	(29.2)	13	(9.6)
Employee																				
1st	123	(10.4)	11	(8.9)	124	(10.5)	9	(7.3)	144	(12.1)	16	(11.1)	138	(11.5)	20	(14.5)	172	(14.3)	12	(7.0)
2nd	101	(8.5)	15	(14.9)	125	(10.6)	11	(8.8)	93	(7.8)	17	(18.3)	109	(9.1)	17	(15.6)	95	(7.9)	9	(9.5)
3rd	150	(12.7)	26	(17.3)	132	(11.2)	15	(11.4)	157	(13.2)	25	(15.9)	144	(12.1)	25	(17.4)	155	(12.9)	11	(7.1)
4th	298	(25.2)	46	(15.4)	276	(23.4)	32	(11.6)	250	(21.0)	29	(11.6)	261	(21.8)	55	(21.1)	227	(18.9)	28	(12.3)
5th	511	(43.2)	87	(17.0)	522	(44.3)	65	(12.5)	544	(45.8)	85	(15.6)	543	(45.4)	104	(19.2)	554	(46.1)	66	(11.9)
Participation																				
No	1374	(77.0)	204	(14.8)	1330	(76.7)	129	(9.7)	1323	(76.7)	160	(12.1) ***	1311	(76.7)	206	(15.7) **	1307	(76.6)	128	(9.8)
Yes	411	(23.0)	64	(15.6)	405	(23.3)	51	(12.6)	401	(23.3)	83	(20.7)	399	(23.3)	91	(22.8)	400	(23.4)	33	(8.3)

* *p* < 0.05, ** *p* < 0.01, *** *p* < 0.001.

**Table 2:** The distribution of study sample cohort with dental sealant in the NHISS by participation.

Variables	3rd Grade (2011)				4th Grade (2012)				5th Grade (2013)				6th Grade (2014)				7th Grade (2015)			
	Subject		with Sealant		Subject		with Sealant		Subject		with Sealant		Subject		with Sealant		Subject		with Sealan	
	N	(%)	N	(%)	N	(%)	N	(%)	N	(%)	N	(%)	N	(%)	N	(%)	N	(%)	N	(%)
Case (participation = yes)																				
Total	411	(100.0)	64	(15.6)	405	(100.0)	51	(12.6)	401	(100.0)	83	(20.7)	399	(100.0)	91	(22.8)	400	(100.0)	33	(8.3)
Gender																				
Boys	219	(53.3)	31	(14.2)	218	(53.8)	28	(12.8)	207	(51.6)	44	(21.3)	203	(50.9)	41	(20.2)	205	(51.3)	21	(10.2)
Girls	192	(46.7)	33	(17.2)	187	(46.2)	23	(12.3)	194	(48.4)	39	(20.1)	196	(49.1)	50	(25.5)	195	(48.8)	12	(6.2)
Insurance type																				
Medicaid	8	(1.9)	1	(12.5)	7	(1.7)	-	(0.0)	8	(2.0)	2	(25.0)	11	(2.8)	3	(27.3)	12	(3.0)	-	(0.0)
Self-employed	131	(31.9)	22	(16.8)	121	(29.9)	10	(8.3)	115	(28.7)	28	(24.3)	107	(26.8)	25	(23.4)	105	(26.3)	6	(5.7)
Employee	272	(66.2)	41	(15.1)	277	(68.4)	41	(14.8)	278	(69.3)	53	(19.1)	281	(70.4)	63	(22.4)	283	(70.8)	27	(9.5)
Income quintile																				
Self-employed																				
1st	11	(8.4)	4	(36.4)	11	(9.1)	-	(0.0)	10	(8.7)	1	(10.0)	15	(14.0)	-	(0.0)	13	(12.4)	-	(0.0)
2nd	11	(8.4)	1	(9.1)	17	(14.0)	3	(17.6)	13	(11.3)	2	(15.4)	10	(9.3)	3	(30.0)	12	(11.4)	1	(8.3)
3rd	38	(29.0)	5	(13.2)	29	(24.0)	1	(3.4)	34	(29.6)	10	(29.4)	21	(19.6)	6	(28.6)	24	(22.9)	2	(8.3)
4th	33	(25.2)	5	(15.2)	28	(23.1)	4	(14.3)	24	(20.9)	8	(33.3)	29	(27.1)	6	(20.7)	27	(25.7)	1	(3.7)
5th	38	(29.0)	7	(18.4)	36	(29.8)	2	(5.6)	34	(29.6)	7	(20.6)	32	(29.9)	10	(31.3)	29	(27.6)	2	(6.9)
Employee																				
1st	38	(14.1)	4	(10.5)	39	(14.2)	4	(10.3)	35	(12.6)	7	(20.0)	31	(11.1)	8	(25.8)	37	(13.1)	2	(5.4)
2nd	13	(4.8)	1	(7.7)	21	(7.6)	7	(33.3)	22	(7.9)	6	(27.3)	32	(11.4)	3	(9.4)	23	(8.2)	2	(8.7)
3rd	27	(10.0)	5	(18.5)	24	(8.7)	10	(41.7)	36	(13.0)	10	(27.8)	33	(11.8)	5	(15.2)	35	(12.4)	2	(5.7)
4th	80	(29.6)	11	(13.8)	78	(28.4)	17	(21.8)	71	(25.6)	12	(16.9)	71	(25.4)	21	(29.6)	66	(23.4)	6	(9.1)
5th	112	(41.5)	20	(17.9)	113	(41.1)	54	(47.8)	113	(40.8)	18	(15.9)	113	(40.4)	26	(23.0)	121	(42.9)	15	(12.4)
Control (participation = no)																				
Total	1374	(100.0)	204	(14.8)	1330	(100.0)	129	(9.7)	1323	(100.0)	160	(12.1)	1311	(100.0)	206	(15.7)	1307	(100.0)	128	(9.8)
Gender																				
Boys	713	(51.9)	113	(15.8)	692	(52.0)	77	(11.1)	690	(52.2)	82	(11.9)	690	(52.6)	93	(13.5) *	690	(52.8)	71	(10.3)
Girls	661	(48.1)	91	(13.8)	638	(48.0)	52	(8.2)	633	(47.8)	78	(12.3)	621	(47.4)	113	(18.2)	617	(47.2)	57	(9.2)
Insurance type																				
Medicaid	18	(1.3)	-	(0.0)	19	(1.4)	4	(21.1)	20	(1.5)	1	(5.0)	20	(1.5)	2	(10.0)	19	(1.5)	3	(15.8)
Self-employed	437	(31.8)	58	(13.3)	401	(30.2)	32	(8.0)	385	(29.1)	40	(10.4)	370	(28.2)	46	(12.4)	358	(27.4)	26	(7.3)
Employee	919	(66.9)	146	(15.9)	910	(68.4)	93	(10.2)	918	(69.4)	119	(13.0)	921	(70.3)	158	(17.2)	930	(71.2)	99	(10.6)
Income quintile																				
Self-employed																				
1st	31	(7.1)	4	(12.9)	33	(8.2)	-	(0.0) *	33	(8.6)	3	(9.1)	33	(8.9)	2	(6.1)	41	(11.5)	4	(9.8)
2nd	73	(16.7)	7	(9.6)	63	(15.7)	3	(4.8)	49	(12.7)	4	(8.2)	46	(12.4)	2	(4.3)	38	(10.6)	2	(5.3)
3rd	87	(19.9)	9	(10.3)	89	(22.2)	6	(6.7)	95	(24.7)	8	(8.4)	86	(23.2)	11	(12.8)	81	(22.6)	4	(4.9)
4th	119	(27.2)	21	(17.6)	100	(24.9)	15	(15.0)	99	(25.7)	11	(11.1)	91	(24.6)	14	(15.4)	92	(25.7)	5	(5.4)
5th	127	(29.1)	17	(13.4)	116	(28.9)	8	(6.9)	109	(28.3)	14	(12.8)	114	(30.8)	17	(14.9)	106	(29.6)	11	(10.4)
Employee																				
1st	85	(9.3)	7	(8.2)	85	(9.4)	4	(4.7)	109	(12.0)	9	(8.3)	107	(11.7)	12	(11.2)	135	(14.7)	10	(7.4)
2nd	88	(9.6)	14	(15.9)	104	(11.5)	7	(6.7)	71	(7.8)	11	(15.5)	77	(8.4)	14	(18.2)	72	(7.8)	7	(9.7)
3rd	123	(13.5)	21	(17.1)	108	(11.9)	10	(9.3)	121	(13.3)	15	(12.4)	111	(12.1)	20	(18.0)	120	(13.0)	9	(7.5)
4th	218	(23.9)	35	(16.1)	198	(21.9)	17	(8.6)	179	(19.6)	17	(9.5)	190	(20.8)	34	(17.9)	161	(17.5)	22	(13.7)
5th	399	(43.7)	67	(16.8)	409	(45.2)	54	(13.2)	431	(47.3)	67	(15.5)	430	(47.0)	78	(18.1)	433	(47.0)	51	(11.8)

* *p* < 0.05.

**Table 3:** Odds ratio (OR) and 95% confidence interval (CI) estimated from panel logistic regression models for dental sealant in the NHISS.

(= Reference)	Unadjusted	Model 1	Model 2
Total			
Gender (= Boys)			
Girls	1.00(0.87−1.15)	1.00(0.87−1.15)	1.00(0.87−1.15)
Insurance type (= Medicaid)			
Self-employed	1.03(0.57−1.85)	1.03(0.57−1.85)	1.06(0.59−1.91)
Employee	1.28(0.72−2.29)	1.28(0.72−2.29)	1.32(0.74−2.36)
Participation (= No)			
Yes	1.35(1.15−1.58) ***		1.35(1.16−1.58) ***
Self-employed			
Gender (= Boys)			
Girls	0.88(0.68−1.15)	0.91(0.70−1.18)	0.91(0.70−1.17)
Income quintile (= 1st)			
2nd	1.08(0.57−2.05)	1.07(0.56−2.02)	1.11(0.59−2.09)
3rd	1.40(0.79−2.48)	1.38(0.78−2.45)	1.39(0.79−2.45)
4th	1.92(1.11−3.34) *	1.89(1.09−3.28) *	1.93(1.12−3.35) *
5th	1.73(1.00−2.99)	1.71(0.99−2.97)	1.75(1.01−3.01) *
Participation (= No)			
Yes	1.63(1.22−2.16) **		1.63(1.23−2.16) **
Employee			
Gender (= Boys)			
Girls	1.04(0.88−1.22)	1.02(0.86−1.20)	1.02(0.86−1.20)
Income quintile (= 1st)			
2nd	1.45(1.00−2.12)	1.45(1.00−2.11)	1.47(1.01−2.14) *
3rd	1.50(1.06−2.13) *	1.50(1.06−2.13) *	1.52(1.08−2.15) *
4th	1.61(1.18−2.21) **	1.61(1.18−2.21) **	1.61(1.17−2.20) **
5th	1.69(1.27−2.27) ***	1.69(1.27−2.27) ***	1.72(1.28−2.29) ***
Participation (= No)			
Yes	1.26(1.05−1.53) *		1.27(1.06−1.54) *

* *p* < 0.05, ** *p* < 0.01, *** *p* < 0.001. Model 1: adjusted for gender and insurance type or income quintile; Model 2: adjusted for gender, insurance type or income quintile, and participation.

**Table 4:** Odds ratio and 95% CI estimated from panel logistic regression models for dental sealant in the NHISS by participation.

(= Reference)	Unadjusted	Model 1
Case (participation = yes)		
Total		
Gender (= Boys)		
Girls	1.05(0.81−1.35)	1.06(0.82−1.37)
Insurance type (= Medicaid)		
Self-employed	1.26(0.49−3.23)	1.29(0.50−3.30)
Employee	1.31(0.52−3.29)	1.33(0.53−3.36)
Self-employed		
Gender (= Boys)		
Girls	0.85(0.52−1.36)	0.89(0.55−1.44)
Income quintile (= 1st)		
2nd	2.08(0.65−6.69)	2.03(0.63−6.55)
3rd	2.17(0.77−6.14)	2.13(0.75−6.03)
4th	2.24(0.79−6.34)	2.16(0.76−6.18)
5th	2.19(0.79−6.12)	2.15(0.77−6.02)
Employee		
Gender (= Boys)		
Girls	1.15(0.85−1.57)	1.14(0.85−1.56)
Income quintile (= 1st)		
2nd	1.03(0.51−2.06)	1.03(0.51−2.07)
3rd	1.26(0.68−2.34)	1.28(0.69−2.37)
4th	1.29(0.77−2.16)	1.30(0.77−2.18)
5th	1.10(0.67−1.81)	1.11(0.68−1.82)
Control (participation = no)		
Total		
Gender (= Boys)		
Girls	0.98(0.83−1.15)	0.97(0.83−1.15)
Insurance type (= Medicaid)		
Self-employed	0.98(0.47−2.03)	0.97(0.46−2.02)
Employee	1.30(0.63−2.69)	1.30(0.63−2.68)
Self-employed		
Gender (= Boys)		
Girls	0.90(0.66−1.23)	0.91(0.67−1.24
Income quintile (= 1st)		
2nd	0.86(0.40−1.85)	0.86(0.40−1.84)
3rd	1.15(0.59−2.27)	1.14(0.58−2.25)
4th	1.85(0.97−3.52)	1.82(0.96−3.47)
5th	1.60(0.84−3.04)	1.59(0.84−3.02)
Employee		
Gender (= Boys)		
Girls	1.00(0.82−1.21)	0.97(0.80−1.18)
Income quintile (= 1st)		
2nd	1.71(1.09−2.69) *	1.72(1.09−2.70) *
3rd	1.68(1.10−2.57) *	1.69(1.10−2.57) *
4th	1.79(1.21−2.65) **	1.79(1.21−2.65) **
5th	2.06(1.44−2.96) ***	2.07(1.44−2.96) ***

* *p* < 0.05, ** *p* < 0.01, *** *p* < 0.001. Model 1: adjusted for gender and insurance type or income quintile.
